# Complete mitochondrial genome of *Prionailurus bengalensis* (Carnivora: Felidae), a protected species in China

**DOI:** 10.1080/23802359.2019.1666678

**Published:** 2019-09-19

**Authors:** Jian-Jun Zhang, Yu-Kang Liang, Zhu-Mei Ren

**Affiliations:** a Shanxi Yangcheng Manghe Rhesus Monkey National Nature Reserve, Yangcheng, China;; b School of Life Science, Shanxi University, Taiyuan, China

**Keywords:** *Prionailurus bengalensis*, Felinae, mitochondrial genome, phylogeny

## Abstract

The complete mitochondrial genome (mitogenome) of the leopard cat *Prionailurus bengalensis* in China was sequenced using the shotgun genome-skimming method. The mitogenome of *P. bengalensis* is totally 17,006 bp in length with a higher A + T content of 60.4% than that of G + C and consists of 13 protein-coding genes (PCGs), two rRNAs, 22 tRNAs, and one non-coding control region. All the PCGs initiate with a typical ATN codon and terminate with a TAA codon except for the four PCGs (*COX1*, *ND2*, *ND3*, and *ND4*) terminating with a single T—and one gene *CYT B* with AGA as stop codon. Most of the tRNA genes have a clover-leaf secondary structure except for *tRNAS* (AGN), which loses a dihydrouridine (DHU) arm. The ML phylogenetic tree showed that *P. viverrinus* nested in the group of *P. bengalensis* individuals, which is close to the clade clustered by the two genera *Otocolobus* and *Felis*.

*Prionailurus bengalensis* (Carnivora: Felidae: Felinae), commonly called leopard cat, is a widespread species, and the distribution range extends throughout Southern, Eastern, and Southeast Asia, which reflects its adaptation to a broad habitat niche (Hemmer 1978; Ross et al. 2015). This species has been listed in CITES Appendix II, endangered species of the Red List of Chinese Species, and also designated as “Least Concern” ver 3.1 by IUCN (Ross et al. 2015). To date, seven complete mitochondrial genomes (mitogenomes) of *P. bengalensis* from South Korea, West China, and Southeast Asia were reported (Park 2011; Tan et al. 2016; Li et al. 2016). We, here, sequenced the complete mitogenome of *P. bengalensis* from north China and constructed its relationship with other Felinae species combined with the data from GenBank.

The muscle material was obtained from a dead adult individual of *P. bengalensis* that was killed by poachers and captured by the forest police at the Manghe National Nature Reserve (112°38′37″E, 35°25′21″N), at the animal herbarium of which the specimen was stored (Voucher No. MB-2018M02). We sequenced the mitogenome of *P. bengalensis* by the shotgun genome-skimming method on an Illumina HiSeq 4000 platform (Zimmer and Wen 2015) and assembled and annotated within Geneious v11.0.3 using the complete mitogenomes of *P. bengalensis* from GenBank as the references. We also performed *de novo* assembly using SPAdes v. 3.7.1 (Bankevich et al. 2012).

The complete mitogenome of *P. bengalensis* is a circular-closed molecule with 17,006 bp in length (GenBank Accession No. MN121632) and consists of 13 protein-coding genes (PCGs; *COX1-3*, *ND1-6*, *ND4L*, *ATP6*, *ATP8*, and *CYT B*), two rRNAs (12S and 16S rRNA), 22 tRNAs, and one non-coding region. Most of the mitochondrial genes are encoded on the H-strand except for one PCG (*ND6*) and eight tRNA genes (*tRNA-Gln*, *tRNA-Asn*, *tRNA-Ser*, *tRNA-Glu*, *tRNA-Ala*, *tRNA-Pro*, *tRNA-Tyr*, and *tRNA-Cys*) on L-strand. The nucleotide composition of the whole mitogenome is 33.0% A, 26.0% C, 13.6% G, and 27.4% T with a higher A + T content (60.4%) than that of G + C (39.6%). All the PCGs initiate with a typical ATG codon expect for *ND2* stating with ATC, and both *ND3* and *ND5* with ATA. Eight PCGs have a typical TAA termination codon, whereas others terminate with a single T—(*COX 3*, and *ND2*, *ND4*), TA– (*ND3*), or AGA (*CYT B*), respectively. We predicted the secondary structure of tRNAs using tRNAscan-SE (Lowe and Chan 2016) and found that 21 tRNA genes had a clover-leaf secondary structure, whereas *tRNAS* (AGN) lost a dihydrouridine (DHU) arm.

We constructed the phylogenetic relationship of leopard cat with other Felinae species using RAxML program with GTRGAMMA model and 1000 bootstrap replicates (Stamatakis 2014). The phylogenetic tree showed that the species *Prionailurus viverrinus* nested in the group of *P. bengalensis* individuals, which is close to the clade clustered by the two genera *Otocolobus* and *Felis* (Figure 1). It is necessary to further examine the monophyly of *P. bengalensis* and taxonomy of the species *P. viverrinus* using more samples and/or morphological and molecular data.

**Figure 1.**
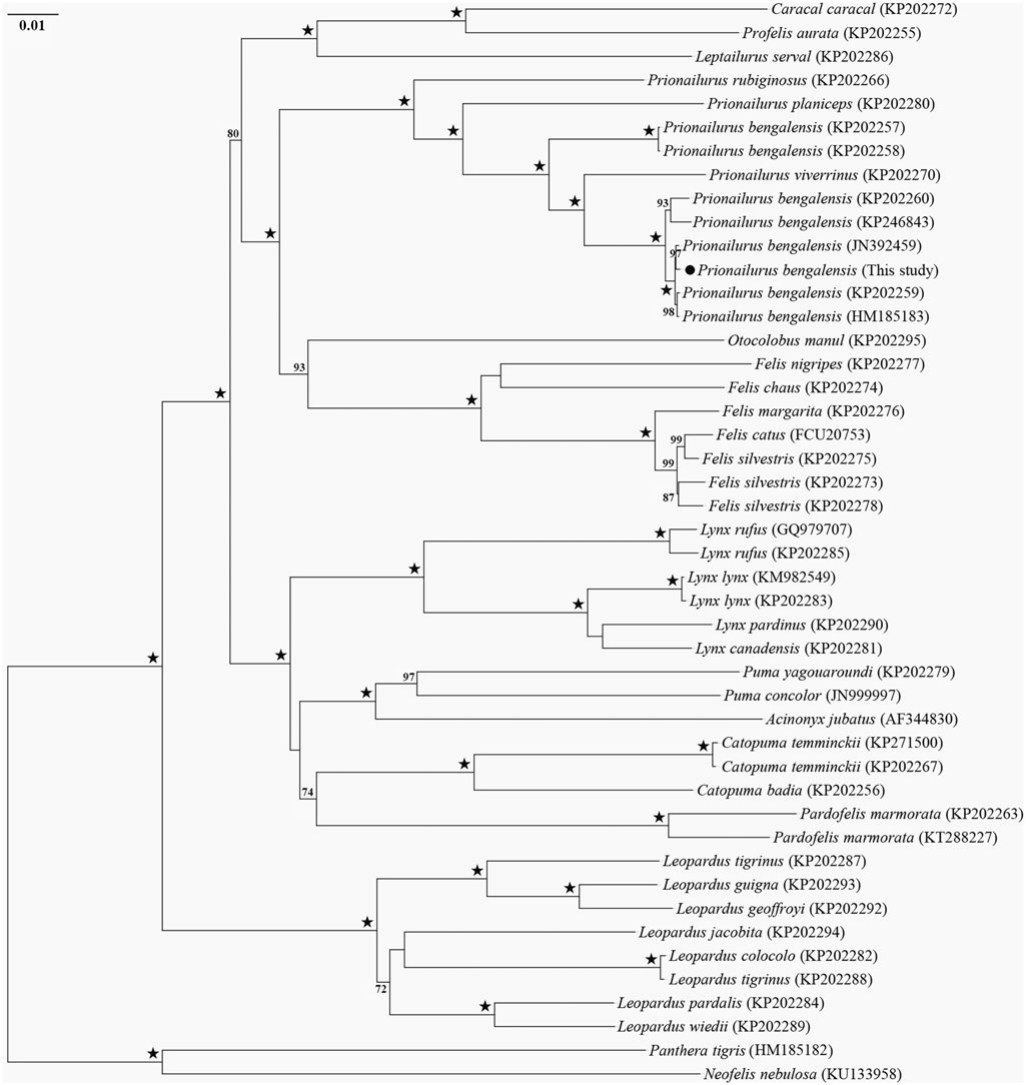
Phylogenetic tree of *Prionailurus bengalensis* and other Felinae species based on 13 protein-coding genes using RAxML program with *Panthera tigris* and *Neofelis nebulosi* as outgroups. Numbers associated with branches are BS >70% and “*” represents nodes with 100% BS.

## References

[CIT0001] Bankevich A, Nurk S, Antipov D, Gurevich AA, Dvorkin M, Kulikov AS, Lesin VM, Nikolenko SI, Pham S, Prjibelski AD, et al. 2012 SPAdes: a new genome assembly algorithm and its applications to single-cell sequencing. J Comput Biol. 19:455–477. 22506599 10.1089/cmb.2012.0021 PMC3342519

[CIT0002] Hemmer H 1978 The evolutionary systematics of living Felidae–present status and current problems. Carnivore. 1:71–79.

[CIT0003] Li G, Davis BW, Eizirik E, Murphy WJ 2016 Phylogenomic evidence for ancient hybridization in the genomes of living cats (Felidae). Genome Res. 26:1–11. 10.1101/gr.186668.114 PMC4691742 26518481

[CIT0004] Lowe TM, Chan P 2016 tRNAscan-SE On-line: integrating search and context for analysis of transfer RNA genes. Nucleic Acids Res. 44:W54–W57. 27174935 10.1093/nar/gkw413 PMC4987944

[CIT0005] Park YC 2011 The complete mitochondrial genome sequence of the Amur leopard cat, *Prionailurus bengalensis euptilurus*. Mitochondrial DNA. 22:89–90. 22040073 10.3109/19401736.2011.624607

[CIT0006] Ross J, Brodie J, Cheyne S, Hearn A, Izawa M, Loken B, Lynam A, McCarthy J, Mukherjee S, Phan C, et al. 2015. Prionailurus bengalensis. The IUCN Red List of Threatened Species. version 2015-3. International Union for Conservation of Nature. e.T18146A50661611.

[CIT0007] Stamatakis A 2014 RAxML version 8: a tool for phylogenetic analysis and post-analysis of large phylogenies. Bioinformatics. 30:1312–1313. 24451623 10.1093/bioinformatics/btu033 PMC3998144

[CIT0008] Tan S, Xu J, Zou F, Peng Q, Peng R 2016 The complete mitochondrial genome of leopard cat, *Prionailurus bengalensis chinensis* (Carnivora: Felidae). Mitochondrial DNA A DNA Mapp Seq Anal. 27:3073–3074. 25629472 10.3109/19401736.2014.1003915

[CIT0009] Zimmer EA, Wen J 2015 Using nuclear gene data for plant phylogenetics: progress and prospects II. Next-gen approaches. J Syst Evol. 53:371–379.

